# Epigenetic modification and a role for the E3 ligase RNF40 in cancer development and metastasis

**DOI:** 10.1038/s41388-020-01556-w

**Published:** 2020-11-16

**Authors:** Junjiang Fu, Li Liao, Kyathegowdanadoddi Srinivasa Balaji, Chunli Wei, Jaehoon Kim, Jiangzhou Peng

**Affiliations:** 1 Key Laboratory of Epigenetics and Oncology, The Research Center for Preclinical Medicine, Southwest Medical University, Luzhou, 646000 Sichuan China; 2 Post Graduation Department of Studies and Research in Biotechnology, Teresian Research Center, Teresian College (Affiliated to University of Mysore), Siddhartha Nagara, Mysore, Karnataka 570011 India; 3 Department of Biological Sciences, Korea Advanced Institute of Science and Technology, Daejeon, 34141 South Korea; 4 Department of Thoracic Surgery, The Third Affiliated Hospital of Southern Medical University, Guangzhou, 510500 Guangdong China

**Keywords:** Cancer genetics, Epigenetics

## Abstract

RNF40 (OMIM: 607700) is a really interesting new gene (RING) finger E3 ubiquitin ligase containing multiple coiled-coil domains and a C-terminal RING finger motif, which engage in protein–DNA and protein–protein interactions. RNF40 encodes a polypeptide of 1001 amino acids with a predicted molecular mass of 113,678 Da. RNF40 and its paralog RNF20 form a stable heterodimer complex that can monoubiquitylate histone H2B at lysine 120 as well as other nonhistone proteins. Cancer is a major public health problem and the second leading cause of death. Through its protein ubiquitylation activity, RNF40 acts as a tumor suppressor or oncogene to play major epigenetic roles in cancer development, progression, and metastasis, highlighting the essential function of RNF40 and the importance of studying it. In this review, we summarize current knowledge about *RNF40* gene structure and the role of RNF40 in histone H2B monoubiquitylation, DNA damage repair, apoptosis, cancer development, and metastasis. We also underscore challenges in applying this information to cancer prognosis and prevention and highlight the urgent need for additional investigations of RNF40 as a potential target for cancer therapeutics.

## Introduction

Cancer is a major public health problem and the second leading cause of death in the world [1–5]. In 2020, almost 2 million new cancer cases and more than 500,000 cancer deaths are predicted in the United States (USA) [5]. Approximately 4.3 million new cancer cases and 2.9 million new cancer deaths occurred in China in 2018—a cancer mortality rate higher than that in the USA [3]. Cancer has been studied extensively for decades, yet the mechanisms underlying its development are not fully understood. Several chromatin-regulated enzymes have been shown to affect posttranslational modifications (PTMs) in histones and nonhistone proteins as well as modifications in DNA. Functional dysregulation of members of this core group of enzymatic factors has frequently been identified as a cause of cancer.

In humans, E3 ubiquitin ligases can be divided into two major families based on the presence of either a really interesting new gene (RING) finger domain or a homologous to the E6-AP carboxyl-terminus domain. About 600 RING finger domain-based ubiquitin ligases have been reported [6]. Various types of RING finger E3 ligases, including RING finger proteins (RNFs), tripartite motifs, MDM2/MDMX (murine double minute 2/4), SCF (SKP1-CUL1-F-box protein) complex, inhibitor of apoptosis proteins, and anaphase-promoting complex/cyclosome, have been reported to play pivotal roles in cancer development, progression, and metastasis [7, 8]. These ligases can tag a target protein with either a single ubiquitin molecule (monoubiquitylation) or chains of multiple ubiquitin molecules (polyubiquitylation).

The *RNF40* gene (also called *BRE1B*, *RBP95*, *Staring*, and *KIAA0661*) encodes an RING finger-type E3 ligase that monoubiquitylates histone H2B at lysine 120. In this review, we summarize the current knowledge of *RNF40* gene structure and function, focusing on its role in H2B monoubiquitylation, DNA damage repair, apoptosis, cancer development, and cancer metastasis. We also highlight challenges in applying this information to cancer prognosis, prevention, and therapy.

## Protein structure, expression, and function of RNF40

**Fig. 1:**
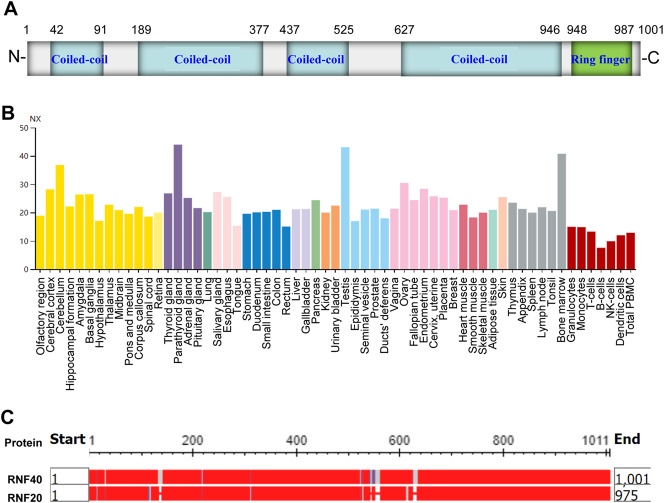
RNF40: domains, expression, and comparison with RNF20. **A** Conserved coiled-coil and RING finger domains in human RNF40. N amino-terminus, C carboxyl-terminus. Numbers indicate the amino acids residues in each domain, adopted from the UniProt database (https://www.uniprot.org/uniprot/O75150). **B** Ubiquitous *RNF40* mRNA expression in humans. RNA-sequencing data were obtained from the Human Protein Atlas (https://www.proteinatlas.org/ENSG00000103549-RNF40/tissue). The method for analysis was as previously described [90, 91]. NX consensus normalized expression. **C** Comparison of RNF40 (GenBank accession number NP_055586.1) and RNF20 (NP_062538.5). Graphical overview by constraint-based multiple alignment tool from NCBI (https://www.ncbi.nlm.nih.gov/tools/cobalt/cobalt.cgi), which shows a column-based method that highlights highly conserved and less conserved columns based on residue’s relative entropy threshold. Alignment columns with no gaps are colored in red or blue, whereas red color indicates highly conserved but blue indicates less conserved. RNF40 and RNF20 have an amino acid identity of 72% and a similarity of 86%.

Using yeast two-hybrid screening, Chin et al. [9] first identified Staring (RNF40) in rats as a syntaxin 1-interacting protein. By searching EST databases, they also found that Staring is a homolog of the human RNF40 protein, which consists of 1001 amino acids with a predicted molecular mass of 113,678 Da. RNF40 contains four coiled-coil domains and a C-terminal RING finger domain, the latter of which is implicated in protein–DNA and protein–protein interactions (Fig. 1A). Northern blot analyses have shown that the *RNF40* gene, which is located on chromosome 16p11.2 in humans, is ubiquitously expressed in human and rat tissues, findings that were also verified by RNA-sequencing analysis in humans (Fig. 1B). Western blot analyses have confirmed the expression of RNF40 protein in the brain of a rat, where it localizes in cytosolic and membrane-related pools [9]. Multiple alternatively spliced isoforms of this protein have also been found in humans (https://www.uniprot.org/uniprot/O75150).

An early study showed that rat RNF40 functions as an E3 ubiquitin ligase in conjunction with Ubch8, an E2 ubiquitin-conjugating enzyme, to facilitate the ubiquitylation and degradation of syntaxin 1, a necessary component of the neurotransmitter-release machinery [9]. Later, Hwang et al. [10] showed that Bre1, a yeast homolog of RNF40, is essential for both histone H2B lysine 120 monoubiquitylation (H2Bub1) and histone H3 lysine 4 methylation (H3K4me). A recent study revealed that liquid–liquid phase separation causes condensation of Bre1 at the other layer of core Lge1 scaffold proteins, and further showed that the resulting layered liquid recruits the E2 ubiquitin-conjugating enzyme Rad6 and the nucleosomal substrate, thereby accelerating the ubiquitylation of H2B [11]. In addition to its role in H2Bub1 and H3K4me modifications, Bre1 protein was also shown by Wood et al. [12] to be essential for histone H3 methylation of lysine 79 (H3K79me) and to exert effects on telomeric silencing and gene expression in yeast. The human homologs of Bre1 are RNF20 (BRE1A, OMIM: 607699) and RNF40 (BRE1B, OMIM: 607700) (Fig. 1C). RNF20 and RNF40 are found to form a heterodimeric complex (RNF20/40 complex) and stabilize each other [13–15]. A size exclusion chromatography analysis suggested that the RNF20/40 complex is composed of four polypeptides: two copies of RNF20 and two copies of RNF40 [14]. RNF20/40 utilizes RAD6 (UBE2A/UBE2B) as an E2 ubiquitin-conjugating enzyme [15] and interacts with WAC (WW domain-containing adapter with coiled coil) to facilitate H2Bub1, thereby impacting cellular functions [16]. The RAP80-binding partner TRAF-interacting protein (or RNF206) was also reported to directly interact with RNF20/40 to regulate the recruitment of DNA damage signaling machinery and promote homologous recombination [17].

H2Bub1 generation is coupled to transcription, which is presumably regulated by chromatin decondensation at transcribed regions. The H2Bub1 modification plays regulatory roles in gene expression, selectively promoting or inhibiting the expression of different subsets of target genes via a mechanism that involves TFIIS and the polymerase-associated factor 1 (PAF1) complex [18] or CRL7^SMU1^ E3 ligase complex [19]. A recent study reported that RNF20/40 plays a role in tumor suppression by regulating p53-dependent gene transcription and mRNA splicing [20]. These subtle regulatory processes specifically affect genes that control genome stability and cell growth, supporting the inference that RNF20 and RNF40 act as tumor suppressors.

## Epigenetic roles of RNF40 in histone modifications and DNA damage

H2Bub1 is an important epigenetic histone modification that regulates gene expression. It is widely accepted that H2Bub1 modifications are positively correlated with active transcription. RNF40 regulates target gene expression in an epigenetic, context-dependent fashion [21]. Because much remains to be clarified regarding relationships among the cancer epigenome, gene expression regulatory mechanisms and DNA repair processes, the roles of H2Bub1 and RNF20/40 in cancer-related chromatin remodeling are of considerable and increasing interest [22].

### Histone H2B monoubiquitylation

Polyubiquitylation, a process in which a target protein is modified to contain multiple ubiquitin moieties, marks proteins for degradation via the proteasome pathway. Recent studies have come to illuminate key cellular roles for monoubiquitylation in this process. Thus, monoubiquitylation plays a pivotal role as one of the largest contributors to histone PTMs, taking its place alongside modifications such as acetylation, methylation, phosphorylation, and sumoylation.

In eukaryotic cells, 146-bp regions of DNA wrap around the core histone octamer containing pairs of H2A, H2B, H3, and H4. H2Bub1 was identified as a central modification of histones that acts in various cellular processes, including transcriptional regulation [23], DNA replication [24], DNA repair [25], maintenance of centromeric chromatin [26], replication-dependent histone mRNA 3′-end processing [27], stem cell differentiation [28, 29], somatic cell reprogramming [30], and heart development [31]. H2Bub1 modifications are lost in genes linked to tumor development, metastasis, and/or poor prognosis in many, but not all, aggressive malignancies [32–35], as discussed in detail below. We will also discuss the involvement of H2Bub1 in DNA DSB (double-strand break) repair. In addition to RNF20/40, multiple E3 ligases, including MDM2 [36] and BAF250b complex [37], are reported to possess H2B ubiquitylation activity; but RNF20/40 is generally considered the major E3 ubiquitin ligase for generating H2Bub1 [14, 15, 25, 38].

The nucleosome imposes a physical barrier that must be overcome during the transcription elongation pause [39, 40]. The following working model for the roles of H2Bub1 in transcription elongation through chromatin has been proposed based on extensive investigations [23]. After transcription initiation, RNA polymerase II (Pol II) pauses upon encountering a nucleosome. This causes optimal recruitment of facilitates chromatin transcription, which, in turn, results in recruitment of the PAF1 complex and H2Bub1 machinery. This is followed by H2Bub1 modifications and effective displacement of one dimer of H2A/H2B from the nucleosome barrier [41, 42]. The resulting partially disassembled nucleosome (hexasome) can then be easily traversed by Pol II. This transcription elongation process is repeated on successive nucleosomes, resulting in an overall increase in transcription efficiency during each round of transcription.

Multiple deubiquitylating enzymes, including USP3, USP7, USP22, USP44, and Eny2, are reported be able to erase H2Bub1 marks and suppress the accessibility of transcription factors and DNA repair proteins to chromatin [43, 44], supporting the dynamic nature of H2Bub1.

### DNA DSB repair

The multiple levels of chromatin organization and its overall complexity underscore the necessity of reorganizing chromatin configurations to facilitate DNA transactions, such as replication, transcription, and repair. Dynamic changes in chromatin condensation and recognition associated with such transactions are accompanied by histone modifications [45, 46]. DNA can be damaged naturally or through environmental factors such as hydrolysis, oxidation, alkylation, and radiation [47]. DNA repair is a corrective process in which a dedicated cellular signaling network, termed the DNA damage response (DDR), temporarily regulates many cellular processes, including metabolism and hydrolysis, in the context of DNA lesions (e.g., DSBs) [18, 48]. The DDR engages the dynamics of protein PTMs, such as ubiquitylation, sumoylation, phosphorylation, acetylation, methylation, and neddylation [49]. The DSB response is mobilized primarily by the protein kinases ATR (ataxia telangiectasia mutated) or ATM (ATM and RAD3 related), which phosphorylate a large number of key players in various repair pathways [50]. Core proteins are committed to the damage response, whereas other cellular processes help meet the challenges of DSB repair. RNF20/40, recently identified as a new component of the DDR network that acts through H2Bub1 modifications, exemplifies this principle [18].

Upon induction of DSBs, ATM phosphorylates Ser172 and Ser553 residues of RNF20 and Ser114 of RNF40, though the central role of ATM is to phosphorylate H2A.X surrounding DSBs [25, 51]. Phosphorylated RNF20/40 is then recruited to DSB sites where it generates DNA damage-associated H2Bub1 modifications, thereby facilitating chromatin opening and increasing accessibility for DNA repair proteins [25]. In this process, repair molecules, including the homologous recombination proteins BRCA1, BRCA2, and RAD51, or nonhomologous recombination proteins Ku80 and XRCC4, are recruited to DSB sites to facilitate successful repair [18, 25, 52].

Like phosphorylated H2AX histones (γH2AX), H2Bub1 modifications largely accumulate proximal to DSBs [53]. H2B lysine 120 (H2BK120) can be either acetylated or ubiquitylated. H2BK120 sites tend to be acetylated within a 1-kb window of the DSB region and are ubiquitylated outside of that window [54]. Recently, So et al. [53] demonstrated that ionizing radiation (IR) induces H2Bub1 modifications via a mechanism that requires both ATM- and ATR-medicated phosphorylation of RNF20/40. Arsenite, a widespread environmental contaminant, was reported to bind to RNF20/40 and inhibit DNA DSB repair [55]. Usp22 and Eny2, components of the SAGA deubiquitylation enzyme complex, are able to remove the ubiquitin tag from H2BK120, a necessary step for class-switch recombination and repair of activation-induced cytidine deaminase and IR-mediated DSBs [43, 56].

### Nonhistone protein ubiquitylation

In addition to H2B, nonhistone substrates of RNF20/40 have been reported, although it cannot be concluded whether RNF20 or RNF40 act through ubiquitylation of such nonhistone proteins to exert their tumor-suppressive or oncogenic functions. RNF20/40 is reported to monoubiquitylate Eg5, a player in spindle assembly during mitosis [57], and eEF1BδL, a heat shock transcription factor [58]. Moreover, RNF20 was found to polyubiquitylate Ebp1, an ErbB3 receptor-binding protein, but it is unclear whether RNF40 is involved in the process [59]. Rat RNF40 protein has also been shown to polyubiquitylate syntaxin 1, which is linked to learning and memory behaviors [9]. Motivated by the expectation that additional nonhistone target proteins of RNF20/40 exist, we are currently seeking to identify EMT-TF-related proteins ubiquitylated by RNF20/40 or RNF40 in breast cancers.

## Role of RNF40 in cancer development and metastasis

Decreases in H2Bub1 and RNF20/40 levels observed in certain advanced cancers, such as colorectal and breast cancers [60], suggest H2Bub1 and H2B ubiquitylation enzymes as cancer markers and novel targets for cancer therapy. RNF40 has been reported as a tumor suppressor in colorectal cancer (CRC), but has also been reported as an oncogene in other cancers, including prostate and liver cancers, and MLL-rearranged acute lymphoblastic leukemia (ALL). The role of RNF40 as a tumor repressor or oncogene will be presented here.

### Breast cancer

The cancer with the highest incidence in women is breast cancer [61, 62]. Tumor-suppressive roles of RNF40 have been reported in breast cancer cells [33]. Consistent with this, the *RNF20* promoter was also reported to be hypermethylated in metastatic breast cancer tumors [63, 64]. H2Bub1 levels were observed to decrease during tumor progression in most malignant and metastatic breast cancers, but were shown to remain high in normal mammary epithelium and benign breast tumors. Key pathways or mechanisms of action of at least some of these changes may be attributable to dysregulation of H2Bub1. In the mammary epithelium, estrogen receptor α (ERα) is a key transcriptional regulator of mammary gland proliferation and differentiation [65]. Compared with ERα-negative status, ERα-positivity is correlated with a more differentiated luminal tumor phenotype and improved survival [66]. A better understanding of the mechanisms involved in ERα regulation may help discover novel targets for treating ERα-positive cancers more effectively. The importance of RNF40-mediated ERα regulation in breast cancer has been investigated in this context. For example, suppressor of Ty 6 homolog (SUPT6H), a histone chaperone and transcription elongation factor [67], was found to be required for estrogen-mediated transcription and maintenance of chromatin structure in breast cancer cells, likely through interaction with RNF40 and regulation of H2Bub1 [68]. It was further shown that SUPT6H protein levels are inversely correlated with breast cancer malignancy. Consistent with this, H2Bub1 and SUPT6H were found to be essential for cell differentiation and suppression of H3K27me3, a repressive histone mark.

Supporting a tumor-suppressive role for H2Bub1 and providing a rational for pursuing H2Bub1-based therapeutic development in breast cancer, Prenzel et al. [33] found that specific inhibition of ERα activity by treatment with bortezomib (or Velcade) caused a global decease H2Bub1, leading to decreased expression of ERα-target genes in MCF7 breast cancer cells. Knockdown of RNF40 has also been shown to reduce ERα-responsive gene transcription and promote estrogen-independent cell survival signaling pathways that support cell proliferation and cell migration. In addition, the proteasome inhibitor Velcade, previously approved by the FDA for the treatment of multiple myeloma and mantle cell lymphoma, was recently reported as a potential inhibitor of ER-positive breast cancer [69].

**Fig. 2:**
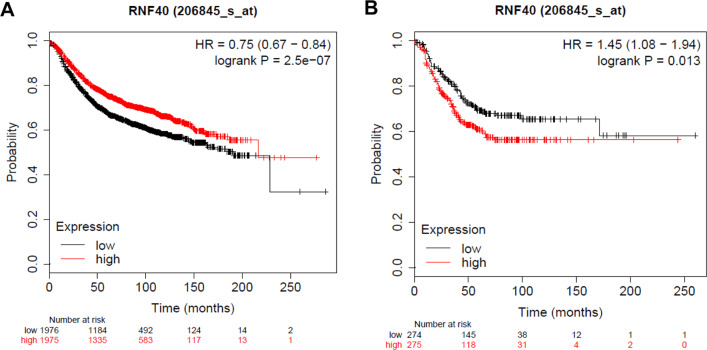
Kaplan–Meier survival analysis according to *RNF40* expression. **A** Kaplan–Meier analyses of relapse-free survival for all-types of breast cancer patients, without regard to subtype, according to *RNF40* mRNA expression. Low *RNF40* mRNA levels were significantly correlated with poor relapse-free survival (*p* = 2.5 × 10^−7^). **B** Kaplan–Meier analyses of relapse-free survival in PR-negative breast cancer patients. High *RNF40* mRNA levels were significantly correlated with poor relapse-free survival (*p* = 0.013). Statistical significance was established as *p* values < 0.05. Kaplan–Meier analyses of survival as a function of *RNF40* mRNA expression [92] were performed using data for 3955 breast cancer patients from an online database (http://kmplot.com/analysis/index.php?p=service&cancer=breast).

Notably, several reports argue for a tumor-suppressive role of RNF40. For example, Duan et al. [57] reported that RNF20/40 functions in spindle assembly and suggested a role for the RNF20/40-Eg5 axis during breast tumorigenesis. These authors found that, during mitosis, RNF20/40 interacts with and monoubiquitylates Eg5, a motor protein critical for spindle assembly in mitosis and tumorigenesis, and this monoubiquitylation facilitates spindle assembly by stabilizing Eg5 protein. Consistent with this, depletion of RNF20/40 in vitro resulted in spindle assembly defects, cell cycle arrest, and apoptosis, whereas depletion of either Eg5 or RNF20/40 in vivo suppressed breast cancer. Significantly, Eg5 and RNF20/40 were concurrently upregulated in breast carcinomas, and higher Eg5 protein expression was correlated with poorer survival of breast cancer patients with luminal A or B type, implicating these proteins as potential targets for cancer treatment and prevention. In support of a pro-oncogenic role for RNF40, a recent random amplified polymorphic DNA analysis demonstrated DNA amplification at the genomic locus of *RNF40* in breast cancer tissues [70]. A Kaplan–Meier analysis revealed that low levels of RNF40 mRNA are significantly correlated with poor relapse-free survival in all breast cancer patients (Fig. 2A), but high levels of *RNF40* mRNA are correlated with poor relapse-free survival in a subtype of progesterone receptor (PR)-negative breast cancer patients (Fig. 2B). These results suggest an oncogenic role for RNF40 in PR-negative breast cancer patients. Also consistent with an oncogenic role, Tarcic et al. [71] demonstrated that downregulation of RNF20 suppressed cell proliferation, tumorigenicity, and metastatic capacity in luminal breast cancer. However, they also observed that knockdown of RNF20 led to opposite effects in basal-like breast tumors. Taken together, these studies suggest that RNF40’s role in tumor development is subtype specific.

### Colorectal cancer (CRC)

CRC is the fourth leading cause of death due to cancer worldwide [72]. H2Bub1 and RNF20/40 levels are frequently decreased in human CRCs [73, 74]. In these patients, the loss of H2Bub1 was found to be correlated with high cancer grade and poor survival, thus suggesting H2Bub1 as a tumor-suppressive marker [74, 75]. However, Schneider et al. [60] recently reported that loss of RNF40 and the accompanying decrease in H2Bub1 resulted in diminished proliferative potential and decreased induction of several NF-κB target genes in CRC cell lines. They further reported that H2Bub1 and RNF40 co-localized in transcribed regions of antiapoptotic genes and that silencing RNF40 robustly increased apoptosis rates owing to an increase in caspase 3/7 activity, a decrease in antiapoptotic proteins, and elevated levels of proapoptotic Bcl-2 protein [60]. Notably, treatment with the caspase inhibitor, Z-VAD-FMK, rescued apoptosis in RNF40-depleted cells. These findings suggest that, by serving as an oncogene that controls the expression of apoptotic genes, RNF40 is necessary for the maintenance of tumorigenic features of CRC [76].

### Prostate cancer (PCa)

PCa is the second most frequently diagnosed cancer in men and the fifth leading cause of death due to cancer worldwide [77, 78]. The androgen receptor (AR), an important steroid receptor transcriptional factor, plays a pivotal role in PCa development [79]. Both RNF20 and RNF40 can physically and functionally interact with AR and regulate its transcriptional activity in cells. Chromatin immunoprecipitation analyses in LNCaP PCa cells showed that androgen treatment increases H2Bub1 levels in transcribed regions of the AR-responsive *PSA* and *FKBP51* genes. Depletion of RNF40 or RNF20 significantly suppressed LNCaP cell growth owing to decreased expression of cell cycle-related genes. Thus, these studies suggest that RNF40 and RNF20 function as oncogenes, acting via ubiquitylation of H2B or other target proteins to promote proliferation of PCa cells [80].

### Liver cancer

Primary liver cancer is one of the most common malignancies around the world, with the hepatocellular carcinoma (HCC) subtype accounting for ~90% of cases [81, 82]. HCC is reported to be the second most common cause of cancer deaths [83]. Immunohistochemistry analyses have shown that RNF40 expression in tumor tissues is significantly higher than that in surrounding normal tissues and high levels of RNF40 are significantly associated with alpha-fetoprotein and tumor-node-metastasis tumor stage. Moreover, 5-year overall survival and disease-free survival rates were shown to be low in HCC patients with high RNF40 expression [84]. Thus, RNF40 might function as a tumorigenic factor in liver cancer. However, the mechanism by which RN40 promotes advanced HCC is still unclear.

### MLL-rearranged acute lymphoblastic leukemia (ALL)

ALL is an aggressive malignancy in newborn children that is associated with poor outcomes [85]. MLL-rearranged ALL (MALL) exhibits a deregulated epigenome that displays heightened sensitivity to epigenetic perturbators [86, 87]. Strong antileukemic effects of the histone deacetylase inhibitor, panobinostat (LBH589), have been demonstrated in MALL xenograft mouse models [88]. In this latter study, panobinostat treatment resulted in prolonged survival and decreased overall disease burden. Mechanistically, in vitro studies showed that inhibition of the H2B ubiquitylation enzyme machinery and associated depletion of H2Bub1 are responsible for the antileukemic activities of panobinostat. Knockdown of WAC phenocopied the loss of H2Bub1 and was accompanied by the induction of cell death. Collectively, these results suggest that, through inhibition of multiple epigenetic pathways, the highly efficacious targeting actions of panobinostat have potential in the treatment of MALL.

### Other cancers

Loss or mutation of CDC73 (cell division cycle 73) found in parathyroid carcinoma was reported to disrupt physical interactions between the PAF1 complex and RNF20/40 and thus lead to loss of H2Bub1 [34]. A cohort analysis of high-grade serous ovarian cancers (HGSOC) revealed global H2Bub1 loss in 77% of tumor patients at all stages (I–IV) [22]. However, loss of RNF20 or H2Bub1 was found in only 6% of primary HGSOC patients. Similarly, it was found that germline BRCA1 mutations are not associated with the global loss of H2Bub1 [22]. Thus, the authors of this study concluded that the regulation of H2Bub1 levels—whether by RNF20, RNF40, BRCA1, or other factors—is complicated. Aberrant expression of different histone-associated “writer” or “eraser” enzymes is most likely responsible for the H2Bub1 loss in HGSOC.

## Challenges and future considerations

RNF20/40 is considered to be the major E3 ubiquitin ligase that catalyzes H2Bub1; however, whether RNF20/40-mediated H2Bub1 is fully responsible for various cancer phenotypes is not fully understand [25]. Physiologically, such as during normal development, RNF20/40 might respond to H2Bub1-dependent DNA damage repair, whereas pathologically in cancers, RNF20/40 might act, at least in part, through ubiquitylation of nonhistone proteins to play roles in cancer development, progression, and metastasis.

Abnormal expression of RNF20/40 contributes to genomic instability, suggesting that RNF20/40 might play a role in the initial stage of carcinogenesis [89]. There are no significant differences in H2Bub1 levels among breast cancer, normal mammary epithelium, and benign tumors; however, H2Bub1 levels are significantly decreased in malignant and metastatic breast cancer cells. Indeed, H2Bub1 is required for DNA DSB repair and its loss promotes genomic instability, suggesting a principal role of H2Bub1 as a tumor-suppressing modification. The mechanisms underlying the loss of H2Bub1 in malignant and metastatic cancers warrant further investigation.

RNF40 has been reported as an oncogene in prostate cancer, liver cancer, and MALL. RNF40 might also act as an oncogene in CRC. In breast cancer, however, both oncogenic and tumor-suppressive roles of RNF40 have been reported, raising the question: friend or foe? In this regard, RNF40 might exert both tumor-suppressor and oncogene functions, an issue that deserves to be further studied. Fundamentally, whether H2Bub1, RNF20, and RNF40 predominantly promote or inhibit carcinoma phenotypes might be influenced by the experimental models chosen or specific cell type and/or disease subtype investigated, and these different roles are probably underpinned by specific transcriptional activators. For example, in patients with PR-negative subtypes, high *RNF40* mRNA levels are significantly correlated with worse relapse-free survival (Fig. 2B), demonstrating the oncogenic role of RNF40 in PR-negative breast cancer.

The functions mediated by RNF40 in the context of carcinogenesis and metastasis are currently not fully understood. RNF40 may predominantly act as a tumor suppresser through H2Bub1 modifications during carcinogenesis, but could also act as an oncogene through ubiquitylation of nonhistone proteins, such as Eg5, during metastasis or advanced stages of cancer. By compromising monoubiquitylation activity, deubiquitylating enzymes may also be involved in precisely regulating the proper levels of H2Bub1 [71]. Further studies of RNF20 and RNF40 as potential targets for cancer therapy are warranted and urgently needed.

## Data Availability

Kaplan–Meier analyses of *RNF40* mRNA expression profiles were performed in breast cancer patients from the database (http://kmplot.com/analysis/index.php?p=service&cancer=breast).
